# SmartPort: A Platform for Sensor Data Monitoring in a Seaport Based on FIWARE

**DOI:** 10.3390/s16030417

**Published:** 2016-03-22

**Authors:** Pablo Fernández, José Miguel Santana, Sebastián Ortega, Agustín Trujillo, José Pablo Suárez, Conrado Domínguez, Jaisiel Santana, Alejandro Sánchez

**Affiliations:** 1 IUMA Information and Communications System, Division of Mathematics, Graphics and Computation (MAGiC), University of Las Palmas de Gran Canaria, Canary Islands 35017, Spain; pablo.fernandez@ulpgc.es (P.F.); mxmeater@gmail.com (S.O.); josepablo.suarez@ulpgc.es (J.P.S.); jaisiel@gmail.com (J.S.); alemagox@gmail.com (A.S.); 2 Imaging Technology Center (CTIM), University of Las Palmas de Gran Canaria, Canary Islands 35017, Spain; agustin.trujillo@ulpgc.es; 3 General Administration, University of Las Palmas de Gran Canaria, Canary Islands 35017, Spain; gerente@ulpgc.es

**Keywords:** seaport, 3D visualization, FIWARE, big data, GIS

## Abstract

Seaport monitoring and management is a significant research area, in which infrastructure automatically collects big data sets that lead the organization in its multiple activities. Thus, this problem is heavily related to the fields of data acquisition, transfer, storage, big data analysis and information visualization. Las Palmas de Gran Canaria port is a good example of how a seaport generates big data volumes through a network of sensors. They are placed on meteorological stations and maritime buoys, registering environmental parameters. Likewise, the Automatic Identification System (AIS) registers several dynamic parameters about the tracked vessels. However, such an amount of data is useless without a system that enables a meaningful visualization and helps make decisions. In this work, we present SmartPort, a platform that offers a distributed architecture for the collection of the port sensors’ data and a rich Internet application that allows the user to explore the geolocated data. The presented SmartPort tool is a representative, promising and inspiring approach to manage and develop a smart system. It covers a demanding need for big data analysis and visualization utilities for managing complex infrastructures, such as a seaport.

## 1. Introduction

A seaport is the connection between the coastal environment and the urban structures. It accommodates ships, enabling commerce and person transportation. With the rise of the Internet of Things (IoT) and the availability of new sensor networks, the amount of information gathered from the natural and man-made elements of the seaport has rapidly increased.

The data gathered from the seaport sources must be continuously monitored, in order to organize and control the ongoing activities. Such activities play a key role in the economic and social development of the community, enabling commerce, transportation of goods and people, environmental protection, *etc.*

This important social role is particularly notorious for the seaport of Las Palmas de Gran Canaria. Placed in the Canary Islands, Spain, it is one of the most important seaports of the western coast of Africa, serving as a connecting point between Africa, Europe and America, as well as a major stop for many goods coming from Asia. Las Palmas seaport receives more than 900,000 Twenty-foot Equivalent Units (TEU) [1] containers and almost two million travelers each year. All of these activities are combined with an extensive range of nautical sport activities, a fishing industry and touristic services.

The body entrusted for the monitoring, maintenance and decision-making of this seaport is Las Palmas de G.C. Port Authority [2]. This institution relies on receiving accurate information about the nearby vessels, containers, meteorological data and the state of the sea. Much of this information comes from a network of sensors deployed all over the seaport and its surroundings.

These large volumes of information usually are geolocated, including their management in the field of big geo-data, a particular problem within big data analysis [3]. A computational architecture is therefore needed that enables the Port Authority to collect, store and visualize the data, as well as performing reactive actions based on current readings.

Due to its volume, the aggregation of data collected by a network of sensors during a long period of time needs to be processed to ensure its availability and scalability. The challenges and complexities implicit in big data volumes, their analysis and the importance of their graphic representations are described in the work of Chopade *et al.* [4].

In this scenario, we have recently introduced the SmartPort project [5], a web platform that integrates the tools for the analysis and visualization of the sensor network of Las Palmas de Gran Canaria seaport. In that work, the project was firstly introduced explaining its design and the used technologies.

The SmartPort project is born from a collaboration agreement between the University of Las Palmas de Gran Canaria (ULPGC), Las Palmas de Gran Canaria Port Authority and the FIWARE program. FIWARE is an open project sponsored by the Future Internet Public Private Partnership (FI-PPP) program, created by the European Commission [6]. It enables the validation of new concepts and technologies, as well as new business models and applications.

The goals of SmartPort can be enumerated as:

- Creating a back-end architecture that processes and stores all of the incoming sensor data safely; a data analysis module is also needed to infer meaningful information from the dataset, enabling the efficient storage and retrieval of information for its subsequent processing.
- Implementing high-level features over the data provided by the meteorological and sea sensors, that turn SmartPort into a Decision Support System (DSS); these features must have a positive impact on the decision-making of the Port Authority, offering reactive notifications on the sensors readings.
- Developing a Rich Internet Application (RIA) as the project front-end, providing tools to manage and visualize the sensor data and quick access to current and historical readings.

### Related Work

Decision support systems are a technological tool that aims to improve the operability of many human organizations. These intelligent applications are often labeled as “smart” [7] due to the high level of automation of their tasks.

The inclusion of DSSs in many contexts is a growing study area in which extracting the visual significance of big data volumes is still challenging. In this regard, the survey offered by Zhang *et al.* [8] is noteworthy, as well as the proposals of McCann [9] of a web-based visualization service aimed at the monitoring of oceanographic data. The visual representation of large sensor networks has also been addressed by Talukder [10] for different infrastructures.

For maritime environments, the automation of decision-making is also a current trend of technological progress. Thus, the literature offers many examples of the inclusion of intelligent systems on sea studies, maritime navigation and seaport control.

Nowadays, we can find long-term projects that support data gathering and integration of maritime sensor networks. Incoming data gathered from mareographs are integrated with the measurements of other sensors to provide a reliable model about the state of the sea.

Some of these projects require a data transfer rate that imposes the use of cabled sensors. This is the case of the North-East Pacific Time-series Undersea Networked Experiments (NEPTUNE) project [11], which also implements a *Data Management and Archive System* (DMAS) with similar goals as our proposal. However, most of these systems, including the SmartPort project presented here, rely on wireless sensor networks [12] placed on top of maritime buoys. This kind of data transmission is in itself an ongoing challenge [13] in which the impact of the sea conditions should not be underestimated, as explained by Albaladejo *et al.* [14].

The Port Authority also deals with more domain-specific tasks. One of the most studied specific problems in the context of a merchant seaport is to plan the arrival, storage and transshipments of goods. This transportation is mostly done through a standard system of cranes and containers whose scheduling has been the object of mathematical studies, such as the one proposed by Murty *et al.* [15].

Due to its economic relevance, there are numerous proposed DSS to automatically compute the optimal route that each vessel and container should follow. These systems are based on different strategies, such as *Multi-Criteria Decision Making* (MCDS) agents [16], numerical simulations [17] or evolutionary algorithms [18].

Despite the applicability of the mentioned systems, they generally do not integrate outer information sources, such as environmental or marine traffic data. Moreover, platforms for the acquisition of maritime sensor data like NEPTUNE do not focus on any GIS (Geographic Information System) 3D visualization interface, showing the docking area just as a schematic layout. In this regard, SmartPort offers a solution in which it is possible to integrate sensors’ data from different domains, being an informative tool for the Port Authority.

The planning of fleet routes is often carried out manually by domain experts. However, there are already automatic solutions, like the *TurboRouter* system [19], which offers a generic GIS approach. Such systems could easily make use of the SmartPort interface to display the desired vessel routes. In this regard, some opportunities for the planning of vessel locations are explored in Section 7.

Similarly, on-board navigational systems offer a wide range of outcomes that may be tracked. These systems [20] position the vessel by integrating several environmental parameters and navigational inputs, normally including a *Global Navigation Satellite System* (GNSS). A formal approach to the uncertainty inherent to such sensors can be found in the work of Borkowski [21]. SmartPort integrates current navigational vessel data, being a possible extension of such DSS.

In this work, we go into further detail explaining the FIWARE architecture and modules used in the project (Section 2) and the seaport data sources (Section 3). Hereinafter, the back-end and front-end architectures developed for SmartPort are described (Section 4 and Section 5, respectively).

Throughout the paper, we also highlight the role of the data gathered from meteorological stations and vessel systems. The current sensor network of Las Palmas seaport provides detailed maritime data on a per-minute basis.

However, raw data must be processed to get meaningful insight. This information allows the port technicians to implement strategies for the best use and improvement of the port facilities. Section 6 presents an instance of data analysis applied to the sensor readings received from two different buoys located in the seaport, which addresses the effectiveness of the port docks for containing the swell. Such an analysis could be applied to other environmental parameters, for instance the wind information. These data, in combination with the ship information, can be used to offer a better understanding of the seaport to the Port Authority.

Finally, Section 7 proposes a particular application of SmartPort addressing the calculation of risks on the vessel positions. The proposed DSS uses different parameters regarding the sea status and the vessel locations, integrating both data sources in a single *Fuzzy Inference System* (FIS).

## 2. FIWARE at a Glance

FIWARE is a royalty-free and open source project aimed at creating a sustainable ecosystem for understanding opportunities arising from the new era of digitization caused by the integration of the latest Internet technologies [22,23]. The project is funded by the European Commission under its Future Internet Public Private Partnership (FI-PPP) program, where cooperation with private companies in the technology sector for the development of Internet-based technologies occurs.

The FIWARE platform provides many technological resources. FIWARE is based on elements called Generic Enablers (GEs), which offer reusable and shared modules that cover a multiplicity of usage areas across various sectors [24].

Generic enablers are considered as software modules that offer various functionalities along with protocols and interfaces for operation and communication. The core platform provided by the FIWARE project is based on GEs linked to the main FIWARE Technical Chapters [25].

### 2.1. Generic Enablers Used in SmartPort

The back-end architecture of the project is mainly based on two different modules provided by FIWARE technology: *Orion Context Broker* and *Cosmos*.

#### 2.1.1. Orion Context Broker

A *Context Broker* (CB) allows the management of the whole context information life cycle, including registrations, updates, subscriptions and queries. Using the context broker, it is possible to store the context elements and manage them through updates and queries. Besides, a CB user is able to subscribe to context information. In that case, whenever a condition is triggered (*i.e.*, the context elements have changed or a predefined time has already passed), the user receives a notification.

In this kind of architecture, a component between the context consumers (*i.e.*, smartphones) and producers (*i.e.*, sensors) is needed. The context broker fills that requirement in the previous setup.

The CB accepts two publish/subscribe modes, *Push* and *Pull*. From the perspective of a context producer, the Push method consists of sending the information to the broker as soon as it is available. The user does not need to request this information continuously. On the other hand, the Pull method consists of sending the information requested by the broker.

A fundamental principle of the context broker is the complete dissociation between context producers and consumers. This means that producers publish data without knowing what, where and when the consumers will consume that information. On the other hand, the context consumers retrieve information without knowing that a context producer is publishing a specific event. As a result, the context broker GE is a bridge that allows external applications to manage IoT events in a simple way, since it hides the complexity of the measurements gathered from the IoT resources, such as sensors.

Orion provides two REST API interfaces: NGSI9 and NGSI10. These APIs allow the following operations:

- NGSI9: register content, discover the context availability, subscribe to context availability, update the context availability and unsubscribe from the context availability.
- NGSI10: update content, query content, subscribe to content, update the subscription and unsubscribe from content.

#### 2.1.2. Cosmos

Cosmos and its ecosystem is the big data storage and analysis Generic Enabler reference implementation (GEri). The big data analysis GE is intended to deploy means for analyzing both batch and stream data. Although the streaming part is still in the road-map, the batch part has been widely developed through the adoption and the in-house creation of the following tools:

- A Hadoop As A Service (HAAS) engine. Apache Hadoop is an open-source software framework written in Java for distributed storage and distributed processing of very large datasets on computer clusters built from commodity hardware.
  All of the modules in Hadoop have been designed with the fundamental assumption that hardware failures (of individual machines or racks of machines) are commonplace; thus, they should be automatically handled by the software inside the framework. The core of Apache Hadoop consists of a storage part (Hadoop Distributed File System (HDFS)) and a processing part (MapReduce).
  Cosmos as GE implements a light version based on a shared Hadoop cluster. The official version is based on OpenStack Sahara, which provides a simple means to provision a data-intensive application cluster (Hadoop or Spark) on top of OpenStack.
- Cosmos GUI and an OAuth2 Tokens Generator for Cosmos REST APIs.
- Cygnus is used as a connector between the Orion context broker and Cosmos. It is based on Flume, a distributed service for efficiently collecting, aggregating and moving large amounts of log data.
  It has a simple and flexible architecture based on streaming data flows. It is robust and fault-tolerant with customized reliability mechanisms and many fail-over and recovery methods. It uses a simple extensible data model that allows for any analytic application.

## 3. Understanding Las Palmas de Gran Canaria Seaport Data Sources

The varied nature of the data coming from Las Palmas seaport required an analysis prior to the design of the components of the system. Therefore, in SmartPort, we have differentiated several information sources from which we have distinguished between dynamic and static data, as is explained below. Through the analysis of the volume, variety and velocity of the incoming data, it has been possible to create an architecture that is an efficient and scalable solution.

### 3.1. Static Data

Static data are those that are considered unalterable over time. For the seaport case, we have digitized static data from different sources, such as Google Earth or references from Spatial Data Infrastructures (SDIs). Some examples of data in SmartPort are infrastructures, hotels, restaurants, Automated Teller Machines (ATMs), bus stops and pharmacies.

The sensors that belong to Las Palmas seaport are placed at fixed locations. This allows us to statically store their coordinates, in contrast with more advanced models, such as Mobile Wireless Sensor Networks (MWSNs) [26], that require a more complex control logic.

### 3.2. Dynamic Data

On the other hand, dynamic data are those with temporal variance, usually presenting an update frequency. Thus, they are one of the main sources for big data volumes. An example of dynamic data is those gathered by buoys and meteorological sensors with a refresh rate of 3 min.

We can find several devices that supply us with varied datasets:

1. Meteorological sensors: The information is provided by Geonica 41001, Geonica 05106 and Geonica 52203 sensors; which provide us with the following data: temperature, wind speed and direction, gust magnitude and direction, rainfall, pressure, trend and humidity.
2. Current meter sensors: The information is provided by Aanderaa Instruments series 3791–3798 and Geonica Datamar 2000C; which provide us with the following data: significant spectral wave height (meters), upper third higher waves average height (meters), 10% of maximum wave average height (meters), maximum wave height (meters), average wave period (seconds), wave peak period (seconds), wave pitch average period at point zero (seconds), wave pick average direction (clockwise arc degrees), scattering of wave direction at peak power (clockwise arc degrees), average wave incoming direction (clockwise arc degrees), directionality index (custom indicator), water column pressure above the Acoustic Waves and Currents (AWAC) sensor (decibars), surface orbital speed above the AWAC sensor (meters per second), surface current direction above the AWAC sensor (clockwise arc degrees) and energy density spectrum for time series (spectral band).
3. Ships: The information is provided by the AIS (Automatic Identification System), including vessel parameters, such as: name, length, nationality, operation type, berth quay, company, arrival date, source port, port code, ship type, cruise transit, Lloyd code, departure date, bollards, call sign and destination port.

## 4. SmartPort Back-End Architecture

In this section, the different back-end modules of the SmartPort project are explained. The main goal of these modules is to store data streams coming from the sensors.

Starting with the static data, where a complex architecture is not required, they mostly need a simple storage architecture. That is the case of Relational Database Management Systems (RDBMS).

**Figure 1:**
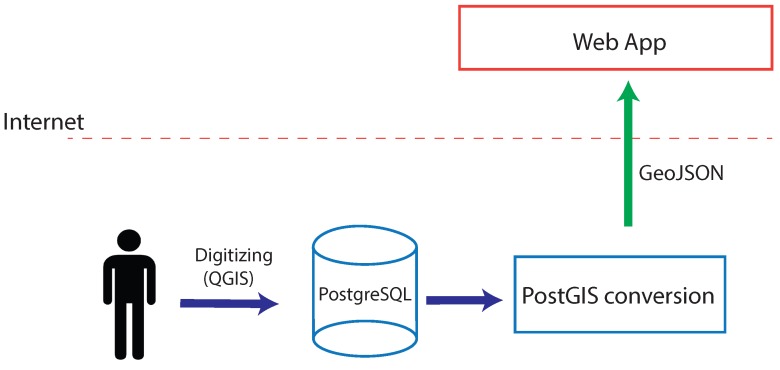
Static data in SmartPort.

The work-flow (see Figure 1) when dealing with static data is as follows:

1. Data are digitized by an operator using a Geographic Information System (GIS). In our case, we have used Quantum GIS (QGIS) [27], a user friendly Open Source GIS licensed under the GNU General Public License.
2. The same GIS edits and stores the data in an RDBMS. The chosen RDBMS has been PostgreSQL with PostGIS, its extension for geographic data management.
3. The previously-stored data are converted into GeoJSON format in order to display them in the viewer. GeoJSON is a format for the encoding of different geographic data structures, which supports the following geometry types: *Point*, *LineString*, *Polygon*, *MultiPoint*, *MultiLineString* and *MultiPolygon*. The geometry lists are represented by a *GeometryCollection* type.

One of the main features of the dynamic sensor data provided in Las Palmas seaport is the refresh rate. It has a maximum value of three minutes due to the nature of its implementation. This implies the fast growth of the information stored in the database, which implies that a traditional database does not satisfy the requirements of this project.

Here is where FIWARE comes into play. It has eased the implementation of new architectures due to its modular interface with the GEs. One of its biggest advantages is the decrease of time dedicated to installing and configuring the system.

In order to obtain the last readings from every sensor, we use the Orion GE. Orion provides a sensor subscription system, which allows us to store and query the last available data. An abstraction layer was made on top of Orion in order to simplify the HTTP transactions and to allow a more agile interaction with the web application. In this way, we can generate simplified data queries via AJAX requests, thereby reducing the amount of calculations done in the client.

Thanks to the previous abstractions and interfaces, a simple query allows us to retrieve data from the sensors. An example to obtain data from the ships would be:

http://<<IP>>:<<Port>>/orion/query.html?sensorType=ship&sensorID=.&pattern=true

Considering that one of the main goals for SmartPort is to obtain sensor historic data, we use Cosmos GE in order to accomplish it.

**Figure 2:**
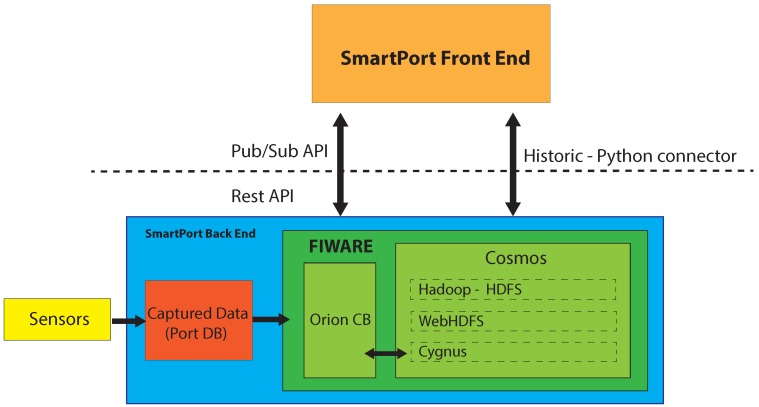
SmartPort back-end architecture.

In our application, as seen in Figure 2, we store the existing data in Orion and afterwards in Cosmos. In order to achieve this connection, we use Cygnus, which is a distributed and reliable data stream channel.

One of the reasons to adopt a big data module is to ensure scalability over time. Currently, the Port Authority of Las Palmas de G.C. is planning to extend the sensor network to support different projects by installing 30 additional sensors per year in a four-year period, having a total of 124 at the end of 2020.

Table 1 shows the expected evolution of the total amount of collected data at the end of each year. The incoming data per day ratio will raise from 140 Mb/day in the 2011–2015 period to 4340 Mb/day in 2020. Once the installation of the sensors is finished, the expected total amount of data stored by the system will increase to 4241.69 Gb at the end of 2020.

Since Hadoop is aimed at working with big volumes of data, it does not offer its optimal capacities with smaller quantities compared to a relational database management system. One of the handicaps in Hadoop is its low query speed, compared to an RDBMS. In order to overcome this issue, we have proceeded to implement a specific data management architecture based on Lambda Architecture [28] in which we differentiate three different layers:

- Batch layer: where all data are stored using Hadoop. This layer pre-processes the views as soon as data arrive. The data post-processing is delegated to the service layer.
- Service layer: the main role of this layer is to answer the queries efficiently. To achieve this, this layer caches the query results in an RDBMS.
- Speed layer: One of the main issues to be solved is the data streaming processing. Thanks to this layer, the most recent data can be included in the analysis result while they propagate through the Hadoop distributed system.

Table 2 shows a comparison of retrieval time before and after the use of the Lambda Architecture and with two RDBMS. The use of the Lambda Architecture reduces the query time up to 88% compared to a standard Hadoop implementation (see Table 3).

### Alert Manager for Sensor Data in SmartPort

Alerts are presented as a strategic goal in SmartPort. Data visualization is not the only important aspect of the application; thus, DSS features have been included in SmartPort. Therefore, we have considered it crucial to implement an alert system to aid the decision-making in the seaport. The alert system allows the application to notify the user when a sensor takes a specific value, enabling in this way the control over the sensor system of the seaport.

The alert system work-flow starts with the creation of an alert. This alert is defined by the sensor type, the attribute to monitor and the condition for the alert. Currently, alerts get triggered when a sensor value is below, equal to or above the condition value.

**Figure 3:**
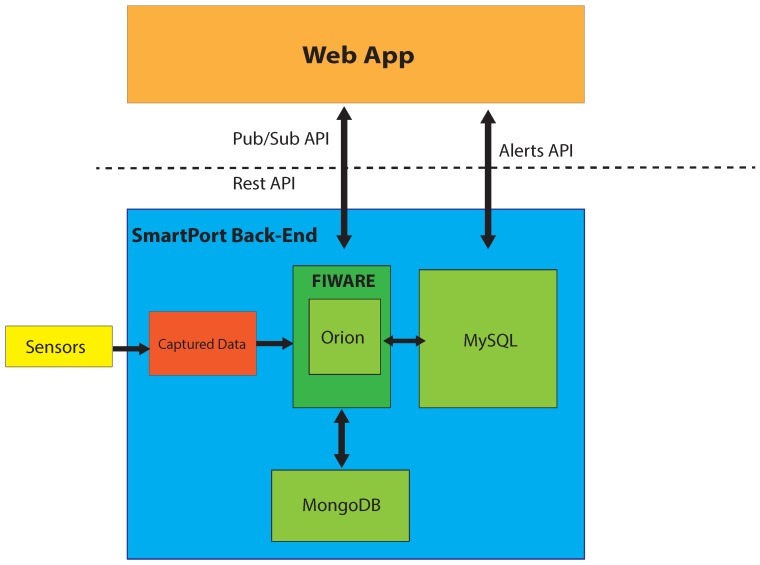
SmartPort alert manager architecture.

When an alert is created, a subscription to Orion is generated using a custom Python API in the back-end. Whenever the condition is triggered, a notification from Orion is sent. When a notification with a condition that raises an alert is received from Orion, it is stored in a MySQL database. Afterwards, the web application fetches the new alert through a custom API, supported by a PHP server module, and displays it (Figure 3). In the front-end, currently active alerts are displayed, as well as alerts that were triggered, but are not active.

## 5. Sensor Data Visualization through SmartPort Rich Internet Application

One of the main goals of SmartPort is to assist the tasks performed by the Port Authority. Therefore, their decision-making processes should be based on reliable and understandable information. Thus, due to the huge volumes of data that their sensor network provides, displaying meaningful information in real-time is quite a challenge.

The front-end of SmartPort is an RIA, which summarizes the seaport data for the user in a comprehensible way. Most of these data reference a spatial location within the seaport of Las Palmas. Displaying the geographical location of the sensors is a common practice that makes the data richer and more understandable, both for indoor [29] and outdoor environments.

For this reason, the RIA is based on a 3D virtual globe, which models the seaport of Las Palmas. This three-dimensional representation of the seaport infrastructures is generated using the Glob3 Mobile framework (G3M). In the next subsections, we explore the main features of this framework and how they have been used to provide a useful tool to the SmartPort end-user.

### 5.1. 3D Visualization of Terrain and Facilities in SmartPort

The 3D scenario of the seaport is the main element of the SmartPort web interface. It provides a meaningful representation of all of the geolocated elements tracked by the SmartPort system, such as sensors, vessels, containers and points of interest. Therefore, the user is able to “virtually” explore the seaport of Las Palmas and its surroundings and to discover the available data sources.

Such GIS visualization falls into the category of virtual Earth engines for the web, which is an open problem by itself in the field of computer graphics. Many map engines can be found to deal with this kind of 3D scenario, such as CesiumJS [30] and WorldWind [31]. A 3D GIS engine creates a dynamic representation of the planet where the developer just has to adjust some parameters of the view and data sources.

During the last few years, we have actively collaborated with the IGO Software company on the creation of G3M, a 3D rendering engine that allows one to create virtual globe applications [32]. G3M offers the web tools needed for visualizing 3D maps, and it is aimed at displaying geo-referenced data [33], allowing the user to virtually explore the seaport and visually locate the sensor stations. As the G3M engine offers all of the functional requirements of the SmartPort front-end [34], it was chosen as the main element of our Internet application.

**Figure 4:**
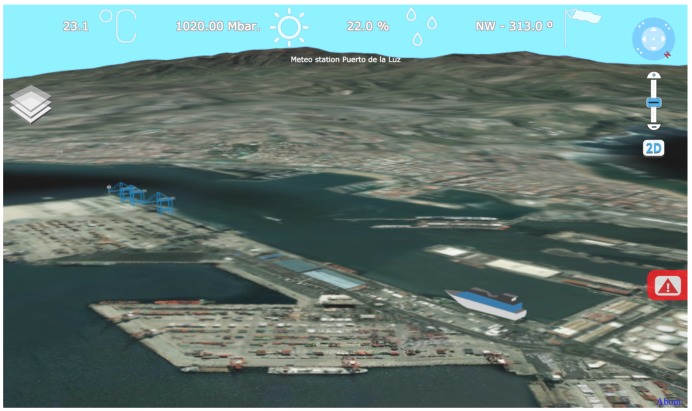
View of the seaport generated on the G3M widget of the SmartPort’s front-end.

G3M is available as a JavaScript library that can be used by the web document. Through a JS API, the developer establishes the main parameters of the virtual globe (imagery, extent, elevation model, *etc.*) and the position of the virtual camera. This virtual scenario is integrated on the web document as a widget that, in the case of SmartPort, is placed as the background of the web taking the whole screen space, as shown in Figure 4.

The JavaScript widget defines an API that is accessible from other modules of the web document. For instance, information of the 3D view, such as the camera position, can be altered through actions on other elements of the web. In a similar way, the G3M widget offers methods that inform the rest of the web about the state of the 3D scenario, so the rest of the interface can be properly updated.

**Figure 5:**
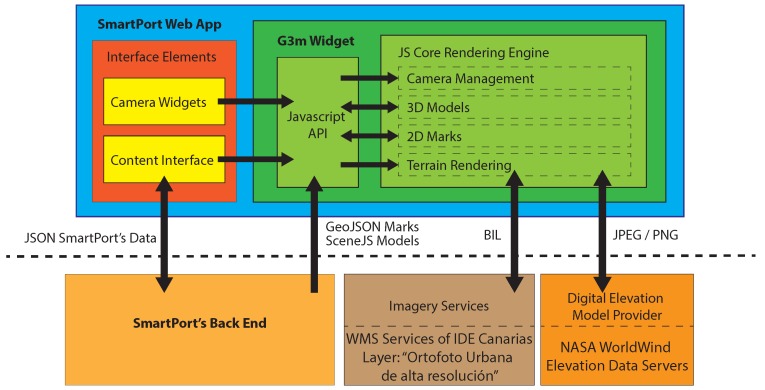
Glob3 Mobile (G3M) integration scheme with the SmartPort front-end and showing access to online services.

The widget can also request information from Internet servers. This capability enables the widget to access not only the large dataset that composes the terrain model, but also to request information from the SmartPort back-end. The whole architecture of the RIA can be seen in Figure 5.

The SmartPort web interface displays a portion of the Earth’s surface that totally covers not only the surroundings of the seaport, but a large portion of sea and the whole island of Gran Canaria. The model of this surface represents a large amount of information, which includes the geolocation of the mesh points, their altitude and the imagery that is displayed on them. In order to manage all of this information, the Earth’s surface is going to be represented by a multiresolution model that evolves depending on the camera position and altitude, according to an algorithm called hierarchical level of detail [35]. This allows the system to fetch only the needed data and to perform a real-time rendering of the surface.

**Figure 6:**
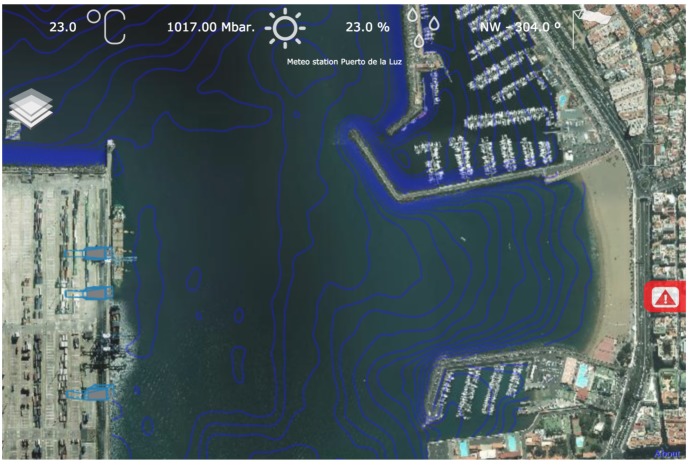
Showing bathymetry layer combined with the orthophoto layer, both fetched using the Web Map Service (WMS) protocol.

Each “chunk” of terrain is formed by a regular grid of triangles covered by a texture. This texture is requested from online Web Map Service (WMS) services and can be composed of several layers. Figure 6 shows how the SmartPort web application combines a bathymetric layer with satellite imagery, to inform the user about the sea depth at every point.

In addition, this surface grid is displaced vertically following a Digital Elevation Model (DEM) that represents the altitude of the surrounding areas of the seaport. This orography, as seen in Figure 4, represents the landscape seen from the seaport, serving as a reference point to locate the user.

G3M also permits the inclusion of 3D objects that are going to be rendered within the virtual Earth scenario. These objects are imported in SceneJS format and can include textures and make use of a global illumination system.

**Figure 7:**
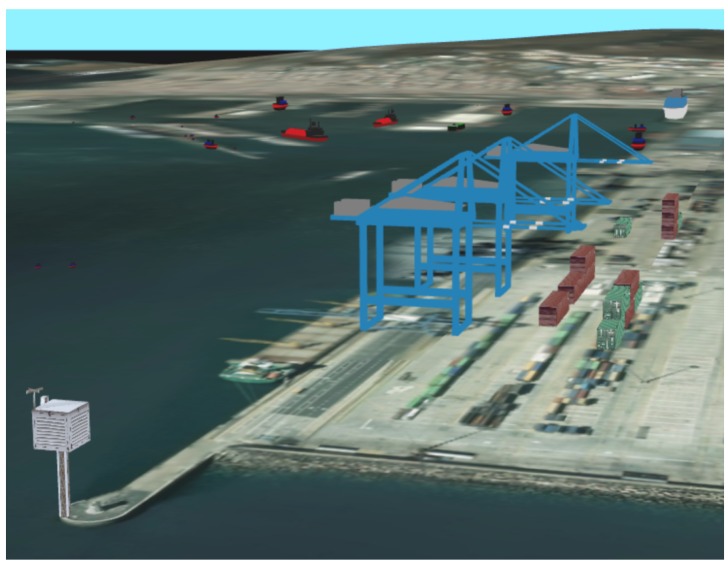
3D models showing the current position of the vessels, containers and cranes within the seaport environment.

SmartPort makes use of this feature to show precisely the location of important items, such as the static data sources (buoys and meteorological sensors) and other movable items. Models of such vessels and cranes can be seen in Figure 7.

One of the utilities integrated on the SmartPort RIA is an option to highlight the location of diverse services related to the seaport activities. These items compound a set that can reach several hundreds of locations.

**Figure 8:**
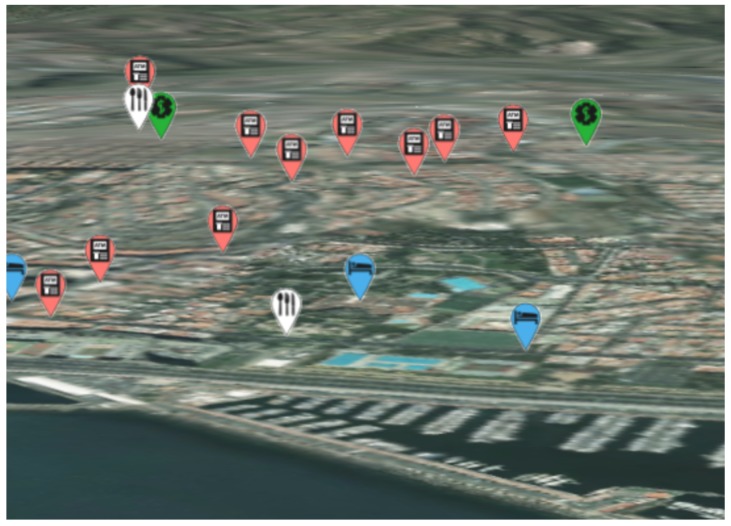
2D billboard marks showing the position of different items on the seaport surroundings.

To show the location of these many items, detailed 3D models cannot be used, as they would cause long rendering times and are scaled by the user perspective. Instead, we use the following rendering solution: for each item, the rendering engine shows a billboard that is projected on the 3D position of that location. The billboard shows a small image and, additionally, some text on it. Billboards are axes aligned within the screen space and do not shrink through perspective projection, as can be seen in Figure 8.

As there can be many items projected on close screen positions, marks could be rendered on top of each other, making them unreadable. New methods for on-the-fly clustering and new kinds of movable marks are topics for upcoming future research.

### 5.2. Rich Internet Application

The main user interface of the SmartPort project has been defined as an RIA based on a 3D visualization of the seaport environment. This 3D environment is then enriched by the inclusion of georeferenced data provided by the SmartPort back-end.

G3M offers a wide range of user-driven interactions that enable the user to move the camera across the virtual scenario. Each one of these user inputs belongs to one of the following groups:

- Desktop mode: classic mouse-and-keyboard interaction paradigm. The user can move the virtual camera by dragging the scenario or combining the mouse movement with keyboard commands that alter the camera pitch and heading. The mouse wheel allows controlling a fast zoom-in/zoom-out camera effect. In addition, the arrow keys allow the user to displace the camera on the geographic space.
- Multi-touch mode: on mobile devices, the user interaction is performed by touching the screen device. G3M recognizes multiple fingers moving on the screen and interprets them as camera movement commands. These multi-touch gestures include double tap (zoom in), single drag (drag scenario), double drag (zoom and rotate effect) and triple drag (pitch and camera heading control).

SmartPort web is mainly intended to be used through the desktop mode, as the web applications normally run on personal computers. However, some users find it more comprehensible to handle on-screen controls, as they offer a visual representation of the camera position and altitude. These kinds of controls are widely used in other GIS applications. For this reason, SmartPort has integrated visual controls on the top-right corner of the screen.

**Figure 9:**
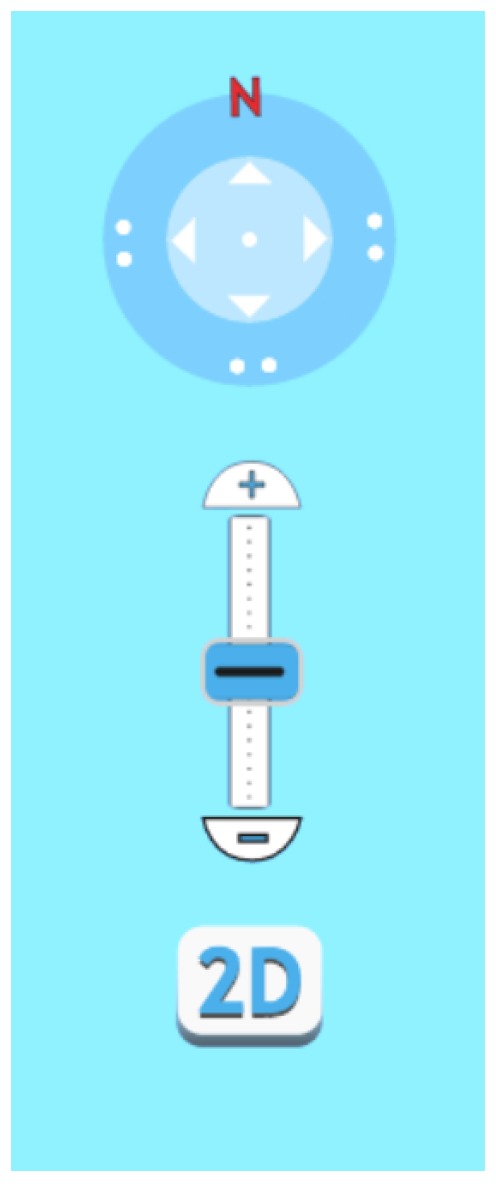
On-screen Head Up Display (HUD) widgets provide control over the virtual camera attitude.

These controls are known as a dragging ball, representing the heading of the camera and its pitch, and a steady scroll that allows one to change the camera distance to the ground. The controls and their use can be seen in Figure 9. These controls are similar to other controls found on well-known virtual globe apps, such as Google Earth [36], easing the user adaptation to the system.

### 5.3. Native App for Mobile Devices

The SmartPort application was initially conceived of to be executed on a computer using an HTML browser. However, it is convenient to use SmartPort on a mobile device, like a tablet. For instance, an operator can navigate to the port and view or edit any of the georeferenced parameters related to the app, such as buoys, sensors, *etc.* Another use case occurs when an alert related to a specific sensor has been raised. In this case, the user sees his or her precise location on the map. As most tablets include GPS sensors, which report the user location, an operator can find the troubling sensor quickly.

Currently, the RIA of SmartPort can be accessed on mobile browsers. However, mobile versions of browsers, included in smartphones and tablets, are not quite efficient at dealing with complex 3D scenes. Besides, the gesture interaction inside the browser canvas does not work properly when using multiple fingers to handle the scene.

In order to efficiently use SmartPort on a mobile device, the best solution is to develop a native version of the application. This is relatively simple using the G3M API, since it is a multi-platform engine. Two main technical tasks have been made to obtain this native version. Firstly, multi-touch gesture handlers have been added to the app to achieve a smooth navigation using the fingers instead of a mouse. Secondly, the native API for each device (iOS or Android) has been used to obtain the real geographical position of the user through the GPS sensor included in the device. In this way, a mark is drawn on the scene indicating that position.

**Figure 10:**
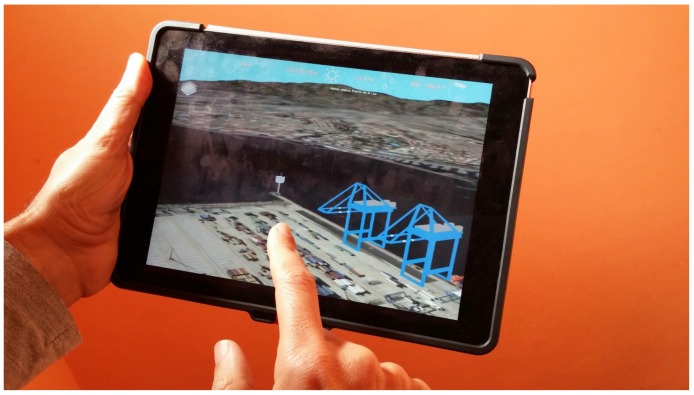
Native version of SmartPort running on an iPad Air 2.

G3M includes level of detail algorithms specifically designed to be run on mobile devices, used on the terrain [37] and other 3D models. This task is needed to obtain a good performance on a mid-range mobile device. Figure 10 shows the app running on a tablet.

## 6. SmartPort Data Analysis

We explore in this section the possibilities of SmartPort as a data analysis system. This analysis is performed on the aggregation of meteorological and maritime information over time, enabling statistical and historical analysis of the data series.

### 6.1. Historical Characterization of Large Datasets

**Figure 11:**
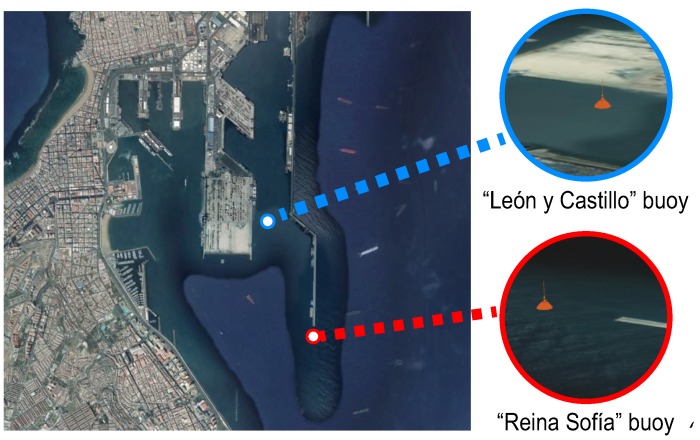
Location of the “León y Castillo” and “Reina Sofía” buoys in Las Palmas de Gran Canaria.

One of the key elements of SmartPort is the data management capabilities. Considering that the analytical part is still being developed, we have already made basic characterization tasks over the stored data in the SmartPort system. This is the case of a dataset taken from two buoys located at the seaport. These buoys have been named “Reina Sofía” and “León y Castillo”, based on their location. The “Reina Sofía” buoy is located at the end of one of the most important docks of the port, exposed to heavy swells, while “León y Castillo” is covered by the seaport itself, as shown in Figure 11.

**Figure 12:**
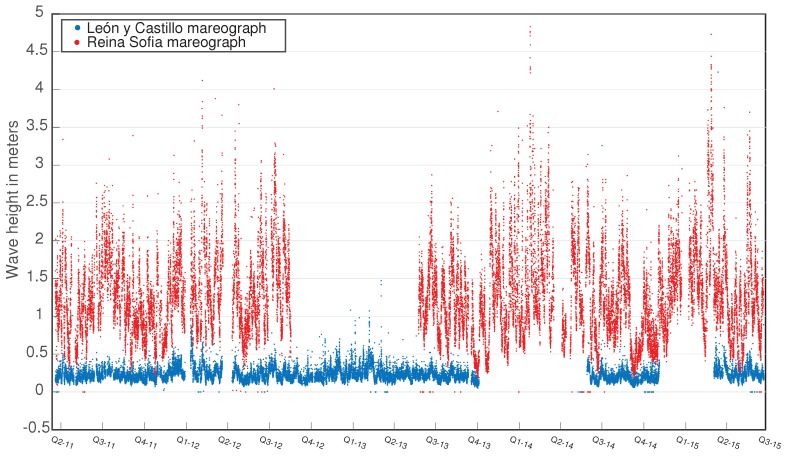
2012–2015 readings of the height of 10% of the maximum wave registered by Mareograph Geonica placed on the “Reina Sofía” and “León y Castillo” buoys.

The first analysis we have made in this setup is the visualization of the whole dataset provided by both buoys, whose locations can be seen in Figure 12. We have chosen the parameter named “10% of maximum wave average height”. It can clearly be seen that the “León y Castillo” readings are notably lower, since the port infrastructure highly smooths the effect of waves. Besides, an oscillatory sequence between different seasons of the year can be observed. This fact is addressed in further detail in Section 6.2. Some missing data can be observed, due to the temporal malfunction of the sensors.

**Figure 13:**
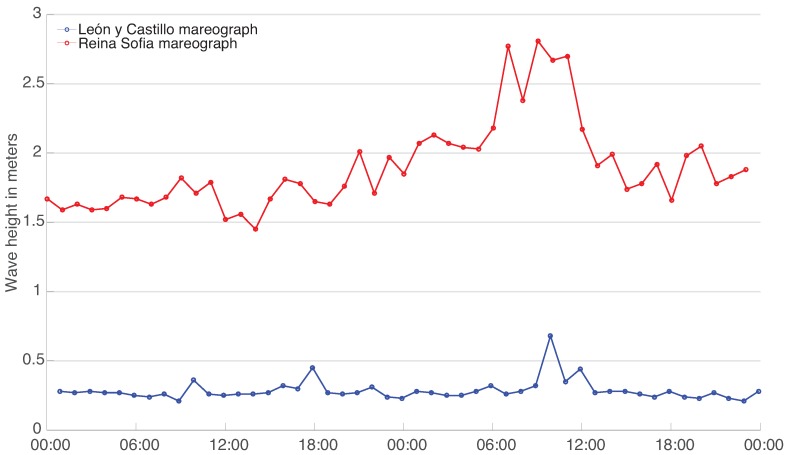
Mareograph Geonica data series of both buoys close-up, centered on days ‘1 July 2014’–‘3 July 2014’.

Additionally, an analysis has been made on a smaller dataset, taking a sample of only one day, as shown in Figure 13.

### 6.2. Seasonal Analysis of Maritime Sensors Output

As was explained in previous sections, SmartPort provides the tools to properly manage huge datasets, regarding the meteorological and sea conditions during long periods of time. As has been stated above, this analysis enables us to perform historical analysis, find trends on the datasets and even extract conclusions about the effects of the different elements of the seaport.

However, in some cases, a temporal clustering of the datasets may allow us to achieve a more meaningful insight on the information gathered by the sensors. In order to achieve this, the SmartPort back-end supports a temporal grouping of the data based on the needs of the study. In this case study, we have considered the dataset described in Section 6.1, and we have arranged the readings of both sensors according to the season in which they were taken. The selection of the clusters used in this study is based on how the state of the sea appreciably varies over the seasons.

**Figure 14:**
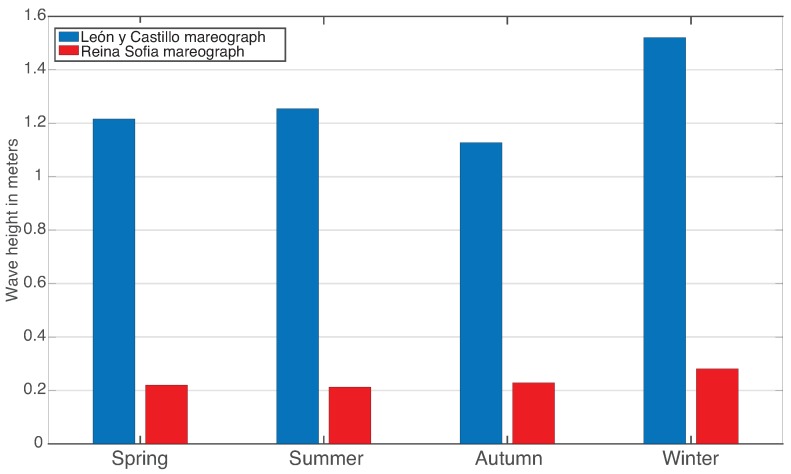
The 10% highest wave’s mean height as registered by Mareograph Geonica on the “Reina Sofía” and ‘León y Castillo” buoys, grouped by season for the years 2011–2015.

In fact, studying the seasonal variance of the mean wave height through the years 2011–2015 allows one to understand that the outer wave increases around 25% from autumn to winter and the inner wave 21% approximately. This seasonal change in the state of the sea can be seen in Figure 14.

Once the variability of the sea conditions across the different seasons of the years has been established, we can extract statistical information about each season from the period 2011–2015. Based on these statistics, the Port Authority may extract meaningful conclusions about the seasonal variance of the swell and the behavior of the sensors in place.

Insightful data about the sensor performance are the presence of readings considered as outliers within this dataset. The criterion used to identify such readings is to consider any data point more than 1.5 interquartile ranges (IQRs) below the first quartile or above the third quartile. The results of such an analysis are presented in Table 4 and Table 5, which show the performance of Mareograph Geonica placed on the buoys of “León Y Castillo” and “Reina Sofía”, respectively.

Table 4 shows the performance of the inner mareograph. It is noteworthy that there is a lack of data for the winter 2013 period, due to a disconnection of the buoy for maintenance. It is also remarkable that, although the buoy offers new data every hour (3600 s), the medium frequency of the data is noticeably higher (around 3900 s). That is due to missing values, lost during the transfer of the signal from the buoy to the port data receiver. The number of outliers is also apparently higher in seasons with higher waves, such as winters and autumns, which could be interpreted as a higher malfunctioning rate of the sensor over those periods.

In a similar way, Table 5 describes the mareograph data measuring the conditions of the sea outside the seaport. The analysis shows that the periods of autumn and winter of 2012 are missing, due to maintenance activities. The medium frequency of the incoming data is 4048 s, due to the greater distance that the signal has to travel. The number of outliers detected in this case has decreased, meaning a more consistent set of readings through each one of the seasons.

**Figure 15:**
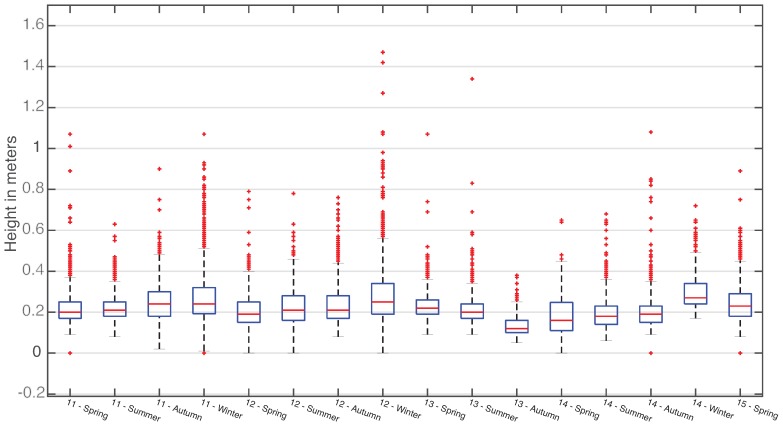
Quartile distribution of the wave height data series from Mareograph Geonica placed on the “León y Castillo” buoy, grouped by season and year.

**Figure 16:**
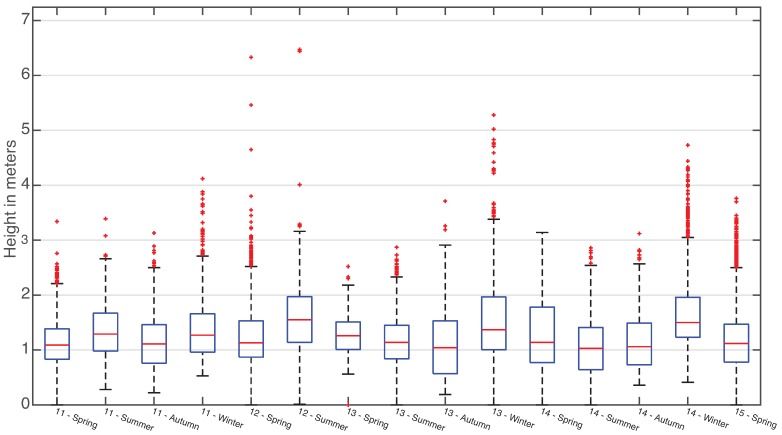
Quartile distribution of the wave height data series from Mareograph Geonica placed on the “Reina Sofía” buoy, grouped by season and year.

Another tool to understand the data managed by SmartPort is statistical plotting of clustered data. In this case, the variance of the data collected through the seasons can be better understood graphically. Figure 15 and Figure 16 are two box plots, centered on the area of greatest point density of both data series. In those figures, it is visible how the concentration of collected data varies accordingly with the expected behavior of the swell of each season. Once more, they accurately show the effectiveness of the dam, reducing the effect of the waves inside the seaport.

## 7. Analyzing Multiple Sensor Data in SmartPort: A Case Study

One of the potential features of SmartPort is the assistance with the relocation and reorientation of ships based on sea condition variables. For this purpose, SmartPort needs a function, named the risk function, which calculates a risk value using as input a set of variables, such as wave height, wave period and current direction. The SmartPort application should evaluate this value with the ship length and orientation and then act accordingly.

**Figure 17:**
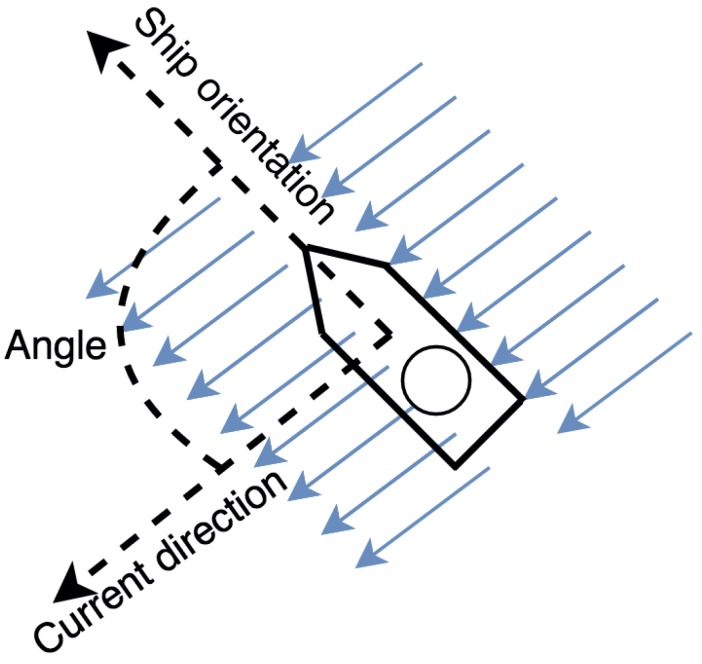
A ship whose angle between its orientation and the current direction is greater than 45 degrees.

For instance, SmartPort should notify that some anchored small ships must be reoriented or relocated if the risk function determines that the risk is medium and the angle between the ship orientation and the current direction is greater than 45 degrees (Figure 17).

In order to create the risk function, we propose to use fuzzy logic. With this approach, we can easily define a system based on rules, and a complex mathematical model is not needed.

One of the fundamental parts of a fuzzy system is the membership grade definition (how much a value belongs to a category) for each magnitude. For this example, we have used trapezoidal functions for risk and wave height magnitudes, while normal distribution functions have been used for the wave period.

**Figure 18:**
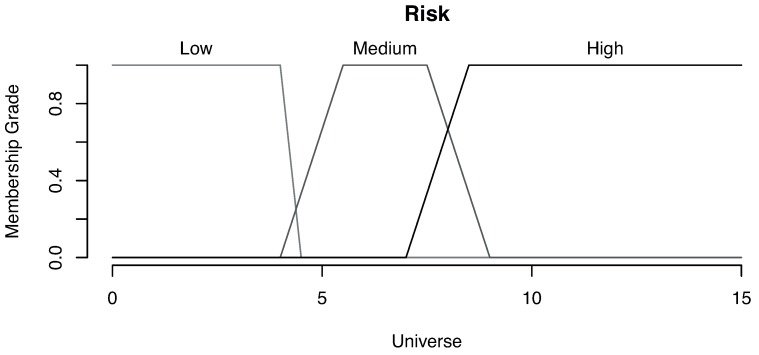
Membership grade for risk.

Figure 18 illustrates the functions that represent the membership grade for each category based on the risk magnitude, showing three distinct categories: low, medium and high. For instance, a risk value is considered low for all values lower than four.

**Figure 19:**
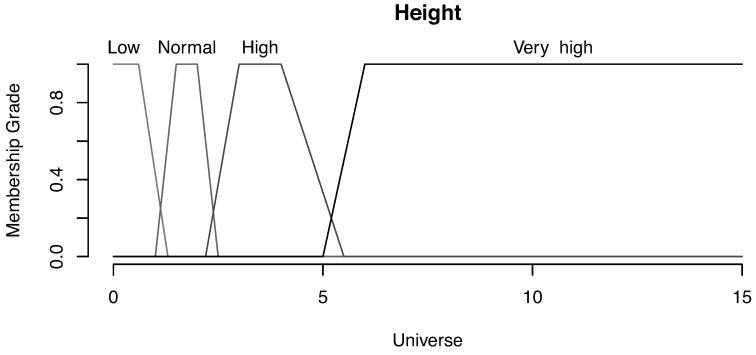
Membership grade for wave height.

In Figure 19, we can see the membership grade for the categories that define the wave height. In this case, we distinguish four categories: low, normal, high and very high. The wave height is considered low for all values smaller than 1.3 meters (with a full membership grade until a value of 0.6 m).

**Figure 20:**
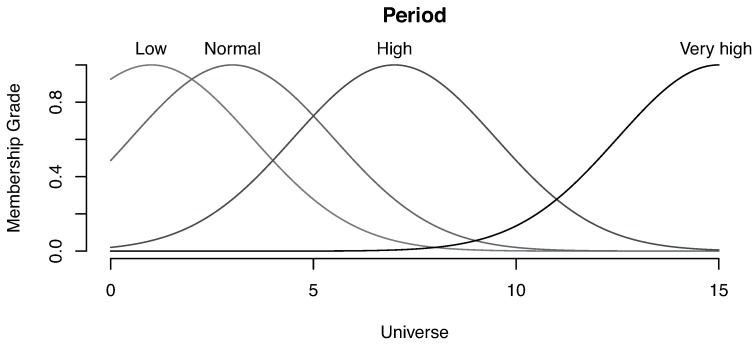
Membership grade for the wave period.

Finally, Figure 20 shows the membership grade for the wave period magnitude. The categories low, normal, high and very high have been defined, and as specified above, normal distribution functions are used to define the membership. For instance, we can see that the low category is defined with a median value of 1 s.

**Figure 21:**
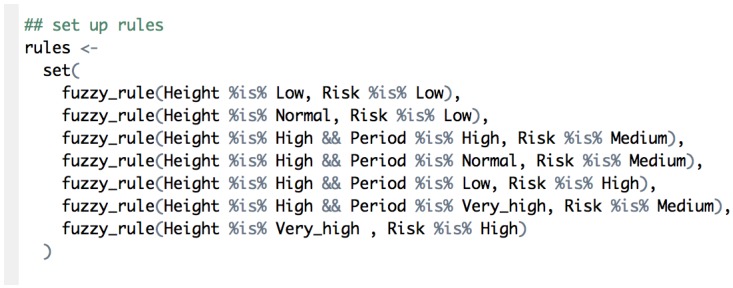
Rule definition.

The other fundamental part of a fuzzy system is the definition of its rules. Using a “natural language”, these rules define the system behavior. Figure 21 shows the rules used in this example. For instance, the first rule of the figure is interpreted as “when the height of the wave is low, the risk level is low”.

**Figure 22:**
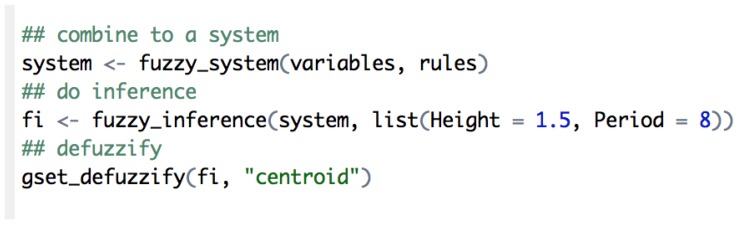
Inference and defuzzification example.

Once the system is defined, we only need to perform an inference with the data read from the sensors and then use the defuzzy function to retrieve the risk value (Figure 22).

Using this simplified system, if we provide as input a wave height value of 1.5 m and a period value of eight seconds, the defuzzy function returns a risk value of 2.1, and no actions are required. However, if we provide a wave height with a value of four meters, the defuzzy function returns a risk value of approximately 6.7. In that case, all small ships that are not aligned with the current should be reoriented.

This case study shows the potential of crossing different sensor data to improve the capabilities of SmartPort as a DSS. Moreover, one of the advantages of the usage of fuzzy logic is its closeness to natural language, which makes it easier to involve port technicians in extending this tool to cover more decision-making aspects of the day-to-day monitoring and management.

## 8. Conclusions and Future Work

In this paper, we have presented SmartPort, showing its main features and demonstrating its utility for the monitoring and the decision-making of a seaport environment.

The analysis of SmartPort is based on the study of the sources of data available to the Port Authority. This study analyzed the seaport environment and many of the measurement systems present in it, mainly buoys, ships and weather stations. Based on the characterization of these sources, we have implemented an architecture that enables us to model the sources of static and dynamic data. An analysis of the data from the sensors present in the port environment has allowed us to implement a system with a proper collection, storage and analysis.

The FIWARE platform has been the mainstay of SmartPort, using software components (generic enablers) as key elements in the development of the back-end of the platform. In this sense, SmartPort stands as a good example of an FIWARE use case. This paper has presented an overview of FIWARE, as well as a detailed analysis of the main components used in the project analysis.

Likewise, the paper introduces an RIA, which enables geolocated data display and control of the different features of SmartPort. Among them, we find the alert system, aimed at improving and easing the decision-making tasks of port elements. This web application makes use of the latest web standards, its main component being a virtual 3D Earth model that represents the seaport of Las Palmas and its surroundings based on the Glob3 mobile framework.

The SmartPort architecture does not only support different datasets offered by the port, but ensures a collection rate that enables one to perform tasks of analysis and transmission strategies to send the data efficiently to the web interface.

One of the lines of future work is to deepen the task of data analysis using new big data technologies. In addition, this analysis module of the project should be based on both statistical techniques and thematic knowledge. This will allow the system to extract meaningful information from the sensor network that would be useful for the port community.

## Figures and Tables

**Table 1:** Expected evolution of total collected data at the end of each year.

Year	Number of Sensors	Incoming Data (Mb/Day)	Total Data Per Year (Gb)	Accumulated Data (Gb)
2011	4	140	49.90	49.90
2012	4	140	49.90	99.80
2013	4	140	49.90	149.71
2014	4	140	49.90	199.61
2015	4	140	49.90	249.51
2016	4	140	49.90	299.41
2017	34	1190	424.17	723.58
2018	64	2240	798.44	1522.02
2019	94	3290	1172.71	2694.73
2020	124	4340	1546.97	4241.70

**Table 2:** Data retrieval time in seconds.

	MySQL	PostgreSQL	Standard Hadoop	Hadoop (Lambda Arch.)
All mareograph data	8.12	9.72	60.03	9.01
One attribute sorted by date	10.55	11.53	74.83	8.91

**Table 3:** Retrieval time performance (%) using Hadoop with Lambda Architecture instead of Standard Hadoop.

	Performance (%)
All mareograph data	85
One attribute sorted by date	88

**Table 4:** The 10% highest wave’s average height of Mareograph Geonica placed on the “León y Castillo” buoy, grouped by season and year.

Year - Season	Min (m.)	Mean (m.)	Max (m.)	Frequency (s)	Outliers	Outliers (%)
11 - Spring	0.00	0.21	1.07	4258.20	130	6.85
11 - Summer	0.08	0.22	0.63	4139.82	147	7.52
11 - Autumn	0.02	0.25	0.90	3771.61	82	4.25
11 - Winter	0.00	0.28	1.07	4509.50	159	9.31
12 - Spring	0.00	0.21	0.79	3647.96	85	5.01
12 - Summer	0.00	0.23	0.78	3923.63	78	3.77
12 - Autumn	0.08	0.24	0.76	3839.16	176	8.79
12 - Winter	0.00	0.28	1.47	3616.78	159	7.40
13 - Spring	0.09	0.23	1.07	3730.14	131	6.15
13 - Summer	0.09	0.21	1.34	3860.20	171	8.13
13 - Autumn	0.05	0.14	0.38	3680.00	20	7.38
14 - Spring	0.00	0.17	0.65	5112.46	33	5.07
14 - Summer	0.06	0.19	3.26	3737.91	117	5.39
14 - Autumn	0.00	0.20	1.08	3699.35	100	10.20
14 - Winter	0.17	0.31	4.23	3615.90	33	7.93
15 - Spring	0.00	0.24	2.09	3745.97	146	6.88

**Table 5:** The 10% highest wave’s average height of Mareograph Geonica placed on the “Reina Sofía” buoy, grouped by season and year.

Year - Season	Min (m.)	Mean (m.)	Max (m.)	Frequency (s)	Outliers	Outliers (%)
11 - Spring	0.00	1.12	3.34	4584.23	72	2.08
11 - Summer	0.28	1.34	3.39	4106.19	41	1.96
11 - Autumn	0.22	1.14	3.13	4077.49	37	5.50
11 - Winter	0.53	1.37	4.12	4596.37	93	6.88
12 - Spring	0.00	1.25	6.33	4635.47	118	3.19
12 - Summer	-9.00	1.59	6.47	4080.98	39	2.90
13 - Spring	0.00	1.27	2.52	3610.99	19	3.52
13 - Summer	0.00	1.18	2.87	3867.58	74	1.51
13 - Autumn	0.19	1.11	3.71	3632.33	32	5.54
13 - Winter	0.00	1.54	5.28	3725.84	111	1.12
14 - Spring	0.00	1.28	3.14	4431.13	17	2.22
14 - Summer	0.00	1.07	2.86	3674.66	49	1.90
14 - Autumn	0.36	1.13	3.12	3649.57	40	9.73
14 - Winter	-9.00	1.65	4.73	4426.20	171	7.32
15 - Spring	0.00	1.20	3.76	3626.52	159	2.08
